# Comparative Evaluation of Novel and Conventional Acid Etchants on the Surface Roughness of Lithium Disilicate and Zirconia Ceramics

**DOI:** 10.1155/ijod/4245703

**Published:** 2026-08-12

**Authors:** Silvia Rojas-Rueda, Franciele Floriani, Fabio Andretti, Hamad Algamaiah, Carlos A. Jurado, Mark Adam Antal, Abdulrahman Alshabib

**Affiliations:** ^1^ School of Dental Medicine, Ponce Health Sciences University, Ponce 00732, Puerto Rico, psm.edu; ^2^ Department of Prosthodontics, College of Dentistry, University of Iowa, Iowa City 52242, Iowa, USA, uiowa.edu; ^3^ Division of Operative Dentistry, Department of General Dentistry, College of Dentistry, University of Tennessee Health Science Center, Memphis 38103, Tennessee, USA, tennessee.edu; ^4^ Department of Restorative Dental Science, College of Dentistry, King Saud University, Riyadh 11545, Saudi Arabia, ksu.edu.sa; ^5^ Department of Operative and Esthetic Dentistry, Faculty of Dentistry, University of Szeged, Szeged 6720, Hungary, u-szeged.hu

## Abstract

**Background:**

Establishing a durable bond to high‐strength dental ceramics, especially zirconia (Zir), is a clinical challenge. This is due to the material’s chemical inertness and its resistance to conventional etching methods. Zir lacks silica, making it unresponsive to traditional hydrofluoric acid (HF) treatments effective for glass ceramics like lithium disilicate (LiDi). This study assesses the efficacy of a novel multiacid etchant in modifying the surface roughness of LiDi and 5 mol% yttria‐stabilized Zir (5Y‐TZP), comparing its performance against three commercial HF‐based etchants.

**Methods:**

Four etchants were tested: Zircos‐E (EDOS [ZiE]), 9.5% porcelain etchant (Bisco [BIS]), 9% porcelain etch (Ultradent [ULT]), and IPS Ceramic Etching Gel (Ivoclar [IPS]). Each was applied for 20 s, 1 min, and 30 min to LiDi (Amber Mill) and 5Y Zir (IPS e.max ZirCAD Prime MT) specimens (*n* = 10 per group). Surface roughness (Ra) was measured with a profilometer. Data were analyzed using two‐way analysis of variance (ANOVA) and Tukey’s honestly significant difference (HSD) test (*α* = 0.05).

**Results:**

On 5Y Zir, Zircos‐E produced significantly higher surface roughness than all other etchants, reaching 0.378 µm at 30 min (*p*  < 0.001), indicating a greater ability to modify the Zir surface topography under the conditions evaluated in this study. On LiDi, it also resulted in the highest roughness (0.785 µm) but with signs of excessive etching and surface degradation. In contrast, the HF‐based etchants produced more controlled and uniform roughening on LiDi, without overetching.

**Conclusions:**

The novel multiacid etchant is highly effective for treating 5Y Zir, creating a favorable microretentive surface for bonding. However, its aggressive action makes it unsuitable for LiDi, where overetching can compromise structural integrity. Selection of etching agents should be material‐specific to optimize clinical outcomes while preserving ceramic quality.

## 1. Introduction

The long‐term success of indirect dental restorations relies on effective surface treatment protocols, which are essential to daily clinical practice. Achieving a durable bond between the restorative material and the tooth structure is paramount for clinical longevity, a concept thoroughly explored in contemporary dental literature. The selection of an appropriate etching technique directly influences the bond strength and, consequently, the prognosis of the restoration. This investigation, therefore, aims to compare the effects of novel and traditional acid etchants on the surface roughness of two widely used all‐ceramic materials: lithium disilicate (LiDi) and 5Y‐zirconia (Zir). By analyzing the resultant surface topography, this study seeks to provide valuable insights into optimizing adhesive procedures for these substrates [1].

LiDi ceramics are commonly used in dental restorations because of their excellent esthetic and mechanical characteristics. Etching with hydrofluoric acid (HF) modifies the surface of LiDi by generating micro‐ and nanoscale porosities, which increase the surface area and expose hydroxyl groups that enable chemical bonding with silane agents. In contrast, Zir‐based ceramics require different surface treatment strategies to improve bond strength and durability. Since Zir is resistant to HF, mechanical methods such as airborne‐particle abrasion (sandblasting) are typically applied to roughen the surface and enhance micromechanical retention [2].

The long‐term success of indirect ceramic restorations largely depends on establishing a reliable and durable bond between the ceramic substrate and the tooth structure [3]. For many years, clinicians have followed well‐validated bonding protocols for silica‐based ceramics such as LiDi [4, 5]. These approaches generally involve etching the intaglio surface with HF [6], followed by the application of a silane coupling agent. HF selectively removes the glassy matrix of the ceramic, producing a microporous surface that enhances micromechanical interlocking with resin cements. Subsequently, silane provides a chemical link between the inorganic ceramic and the organic resin matrix, resulting in a strong and durable adhesive interface [7–9]. Despite its effectiveness, HF presents important clinical limitations. HF is highly toxic and can cause severe soft tissue burns, ocular injury, and systemic toxicity if improperly handled. For this reason, its use requires strict isolation protocols and careful clinical application. Furthermore, some countries have implemented restrictions regarding the availability and use of concentrated HF products. Another limitation is the difficulty of controlling the extent of surface modification once etching has been initiated, as excessive exposure may result in overetching, microstructural degradation, and reduced mechanical properties of ceramic materials. These concerns have motivated the search for alternative surface conditioning strategies that may provide effective surface modification with improved safety and predictability.

To ensure optimal bond strength for LiDi ceramics, it is recommended to use a 10% HF concentration with an etching time of 60 s [10]. This specific protocol has been shown to yield significantly superior results compared to other methods. For CAD/CAM LiDi ceramics, etching with 5% HF for 20 s is also recommended for adequate adhesion. Surface morphology changes with different HF etching protocols include increased surface roughness and porosity, with longer exposure times and higher concentrations producing deeper grooves and irregularities. Specifically, conditioning with 10% HF for 60 s results in a more porous and rougher surface with higher irregularities compared to shorter or less‐concentrated protocols. Excessive etching (e.g., over 60 s) can cause ceramic degradation and weakening, leading to less potentially favorable surface characteristics for future bonding procedures. Conversely, shorter or lower concentration treatments produce less pronounced surface alterations but may still provide adequate adhesion depending on the ceramic type [11]. Currently, most manufacturers recommend etching LiDi ceramics with ~5% HF for 20 s, a protocol that has demonstrated favorable bonding performance while minimizing excessive dissolution of the glassy matrix. Although higher concentrations and longer etching times have been investigated, they may increase surface irregularities and potentially compromise the mechanical integrity of the ceramic. Consequently, the 5% HF for 20 s protocol remains the most widely accepted clinical approach.

The advent and widespread adoption of high‐strength, polycrystalline ceramics, particularly yttria‐stabilized Zir (Y‐TZP), have rendered these traditional bonding methods inadequate [12, 13]. Zir’s superior mechanical properties, like its high fracture toughness and flexural strength, make it a preferred material for monolithic crowns, multiunit fixed partial dentures, and implant abutments [14]. Unlike glass ceramics, Zir lacks a significant silica‐glass phase, making its surface chemically inert and nonresponsive to conventional HF etching [15].

This inherent resistance to chemical conditioning has posed a persistent challenge for adhesive dentistry. In response to these challenges, significant research has focused on developing alternative or adjunctive chemical conditioning methods for Zir. While MDP primers have become the standard of care, the quest for a more effective method to create predictable surface microretention on Zir continues [16]. For Zir ceramics, airborne‐particle abrasion combined with MDP‐containing primers and resin cements is currently considered the gold‐standard bonding protocol. Numerous studies have demonstrated reliable bond strength and satisfactory long‐term clinical performance using this approach. Nevertheless, concerns remain regarding the potential introduction of surface defects and phase transformation associated with mechanical abrasion, which has stimulated ongoing investigation into alternative chemical conditioning methods.

Aggressive sandblasting can induce the tetragonal‐to‐monoclinic phase transformation in Zir, a process associated with volumetric expansion and a reduction in strength. To overcome this, the most common clinical strategy for preparing Zir surfaces has been airborne‐particle abrasion or sandblasting, typically with aluminum oxide (Al_2_O_3_) particles [17]. This technique aims to increase surface area and create mechanical undercuts for resin infiltration. While effective at increasing roughness, sandblasting is a technique‐sensitive and poorly controlled procedure. It can introduce surface flaws, microcracks, and residual stress, which may compromise the long‐term mechanical integrity of the restoration [18].

Recently, a novel multicomponent acid etchant, Zircos‐E, has been introduced, specifically formulated to condition high‐strength ceramics. This solution contains a synergistic blend of hydrofluoric, hydrochloric, nitric, sulfuric, and orthophosphoric acids. Zir’s microstructure makes grain boundaries and the acid‐soluble cubic phase important targets for the first acid attack. Manufacturers state that 15–45 min of etching (progressive pore development) will incrementally roughen the etched surfaces. Thus, testing shorter times than the 1‐h recommended time could simulate interesting scenarios. In addition, the performance of this broad‐spectrum etchant across different classes of dental ceramics has not been fully characterized. Its aggressive nature raises concerns about its potential effects on the more delicate microstructure of silica‐based ceramics [19].

Therefore, this in vitro study aimed to provide a comprehensive analysis of the surface roughness induced by this novel multiacid etchant and three traditional, commercially available HF‐based etchants on two distinct and widely used ceramic materials: a modern high‐translucency 5‐Y Zir and a LiDi glass ceramic. The null hypotheses were as follows: (1) There would be no difference in surface roughness among the four acid etchants on either ceramic type or (2) the etching time would not influence the surface roughness for any group.

## 2. Materials and Methods

Sample size was determined based on previous in vitro studies evaluating ceramic surface roughness after acid conditioning. Ten specimens per group were considered sufficient to detect differences among treatment groups while maintaining feasibility within the experimental design. This in vitro study evaluated the effect of four different acid etchants on the surface roughness of two types of CAD/CAM ceramic materials. A total of 240 specimens were fabricated and divided into 24 groups (*n* = 10 per group) based on the combination of ceramic material, etchant type, and application time. Two commercially available CAD/CAM materials were selected: a LiDi (Amber Mill, Hassbio, Gangneung, South Korea) and a 5‐Y Zir (IPS e.max ZirCAD Prime MT Prime, Ivoclar Group, Schaan, Liechtenstein).

### 2.1. Specimen Preparation

From each material, 120 blocks were sliced to obtain rectangular specimens (10 mm × 10 mm × 2 mm) and sectioned using a low‐speed precision cutting machine (IsoMet Low Speed Precision Cutter; Buehler, Lake Bluff, IL, USA) operating at a cutting speed of 8 mm/min. All specimens were sintered according to the respective manufacturer’s instructions. The surfaces to be etched were then finished with 600‐grit silicon carbide (SiC) paper under water cooling to remove excesses at the edges.

**Figure 1:**
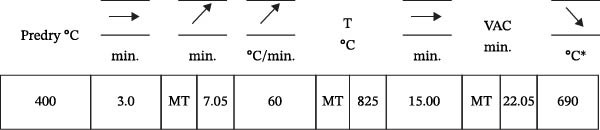
Recommended firing protocol for lithium disilicate (Amber Mill, Hassbio) (Programat CS6, Ivoclar Group) (MT: medium translucency): heating time; min: heating time; heating rate; VAC min.: vacuum time in minutes; °C ^∗^: cooling temperature.

The specimens were fired according to the manufacturers’ instructions (Figure 1 and Table 1). The LiDi material (Amber Mill, Hassbio) can be fired to adjust its translucency. To get a medium translucency (MT) result, the specimens were heated until 825°C.

**Table 1:** Mean zirconia (IPS e.max ZirCAD Prime MT, Ivoclar Group) (Vacumat, Vita) materials tested.

Heating phase	Temperature 1 (°C)	Temperature 2 (°C)	Heating‐up rate (°C/min)	Holding time (min)
Heating phase	20	900	10	–
Holding phase	900	900	–	30
Heating phase	900	1500	3.3	–
Holding phase	1500	1500	–	120
Cooling phase	1500	900	10	–
Cooling phase	900	300	8.3	–

### 2.2. Etching Protocols

The 120 specimens of each ceramic type were randomly divided into 12 subgroups (*n* = 10) for treatment with one of four acid etchants for one of three specified time intervals. The etchants used were as follows:

• Zircos‐E (ZiE): A novel multiacid solution (composition includes HF, HCl, HNO_3_, H_2_SO_4_, and H_3_PO_4_) (EDOS, Brussels, Belgium).
• Bisco Porcelain Etchant (BIS): 9.5% HF (Bisco, Inc., Schaumburg, IL, USA).
• Ultradent Porcelain Etch (ULT): 9% HF (Ultradent Products, Inc., South Jordan, UT, USA).
• IPS Ceramic Etching Gel (IPS): <5% HF (Ivoclar Group, Schaan, Liechtenstein).

The three application times tested were 20 s, 1 min (60 s), and 30 min.

The composition of the multiacid solution is the following: nitric acid (30%–40%), HF (30%–40%), hydrochloric acid (5%–20%), phosphoric/orthophosphoric acid (5%–15%), and sulfuric acid (5–10 %) (information from the manufacturer). The corresponding etchant was applied using disposable applicators to distribute evenly the material and allowed to react for the designated time at room temperature. After etching, all specimens were thoroughly rinsed with a water/air spray for 30 s and then ultrasonically cleaned in distilled water for 15 min to remove any residual acid or crystalline precipitates.

### 2.3. Surface Measurement

Quantitative surface roughness analysis was performed using a high‐precision contact profilometer (Dektak 150, Veeco, Plainview, NY, USA). For each specimen, three parallel traces were made across the center of the etched surface. The profilometer stylus traveled 2 mm for each trace with a constant force of 3 mg. The primary parameter recorded was the average surface roughness (Ra), which represents the arithmetic average of the absolute values of the profile height deviations from the mean line. The mean Ra value for each specimen was calculated from the three traces.

### 2.4. Statistical Analysis

The collected Ra data were imported into statistical software (SPSS v.28.0, IBM Corp., Armonk, NY, USA). Data normality was verified using the Shapiro–Wilk test. For statistical analysis, the experimental groups were organized according to two factors: ceramic material and etchant/time combination. The etchant/time combination factor consisted of the different etching protocols generated by the combination of etchant type and application time. A two‐way analysis of variance (ANOVA) was performed to evaluate the effects of ceramic material, etchant/time combination, and their interaction on surface roughness (Ra). When significant differences were identified, post hoc pairwise comparisons were performed using Tukey’s honestly significant difference (HSD) test. Statistical significance was established at *α* = 0.05.

## 3. Results

**Figure 2:**
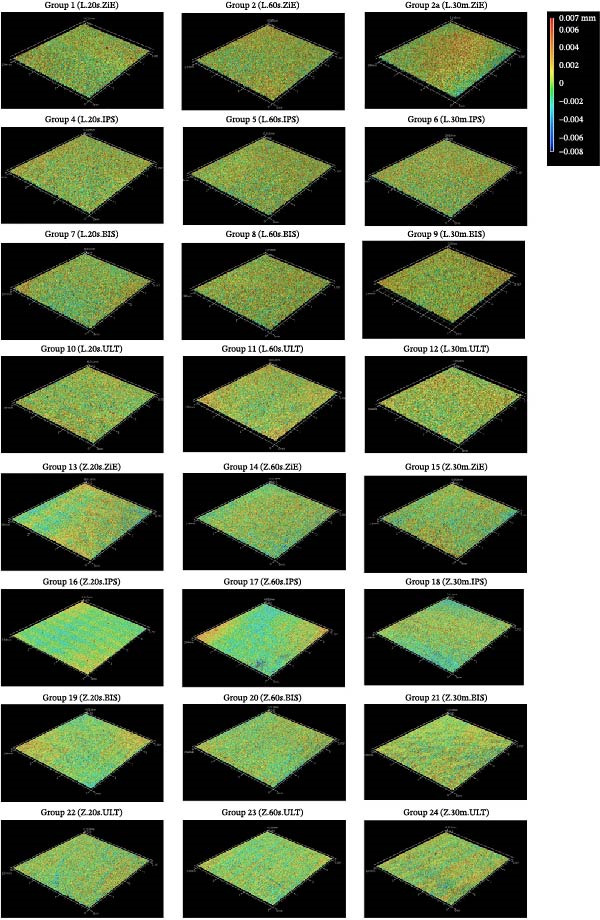
Profilometric 3D images from representative samples of each group. The scale shows positive variation (peaks) (green‐to‐red range) and negative variation (valleys) (green‐to‐blue range). Group 1 (L.20s.ZiE); Group 2 (L.60s.ZiE); Group 2a (L.30m.ZiE); Group 4 (L.20s.IPS); Group 5 (L.60s.IPS); Group 6 (L.30m.IPS); Group 7 (L.20s.BIS); Group 8 (L.60s.BIS); Group 9 (L.30m.BIS); Group 10 (L.20s.ULT); Group 11 (L.60s.ULT); Group 12 (L.30m.ULT); Group 13 (Z.20s.ZiE); Group 14 (Z.60s.ZiE); Group 15 (Z.30m.ZiE); Group 16 (Z.20s.IPS); Group 17 (Z.60s.IPS); Group 18 (Z.30m.IPS); Group 19 (Z.20s.BIS); Group 20 (Z.60s.BIS); Group 21 (Z.30m.BIS); Group 22 (Z.20s.ULT); Group 23 (Z.60s.ULT); and Group 24 (Z.30m.ULT). Li: lithium disilicate (Amber Mill, Hassbio). Z: 5Y‐zirconia (IPS e.max ZirCAD Prime MT, Ivoclar); ZiE: Zircos‐E etchant; BIS: Bisco Porcelain Etchant; ULT: 9% Ultradent Porcelain Etch; IPS: IPS Ceramic Etching Gel; 20 s: 20 s etching time; 60 s: 1‐min etching time; and 30 m: 30‐min etching time.

The two‐way ANOVA revealed that both ceramic material and etchant/time combination had a statistically significant effect on surface roughness (*p*  < 0.001). A significant interaction between these factors was also observed (*p*  < 0.001), indicating that the effect of the etching protocol varied according to the ceramic substrate. Mean surface roughness (Ra) values and standard deviations (SDs) for all 24 experimental groups are presented in Table 2. Representative three‐dimensional profilometric images of all groups are shown in Figure 2.

**Table 2:** Mean surface roughness (Ra, in µm), standard deviation (SD), and Tukey’s grouping for lithium disilicate and zirconia groups.

Material	Etchant	Time	Group	Mean Ra (µm)	Std. deviation	Tukey’s grouping
LiDi	ZiE	20 s	Gr 1	0.574	0.052	g,h
LiDi	ZiE	60 s	Gr 2	0.751	0.075	k,l
LiDi	ZiE	30 m	Gr 3	0.785	0.039	l
LiDi	IPS	20 s	Gr 4	0.535	0.052	f,g
LiDi	IPS	60 s	Gr 5	0.562	0.038	f,g,h
LiDi	IPS	30 m	Gr 6	0.693	0.054	i,j,k
LiDi	BISCO	20 s	Gr 7	0.573	0.032	f,g,h
LiDi	BISCO	60 s	Gr 8	0.671	0.046	i,j
LiDi	BISCO	30 m	Gr 9	0.707	0.068	j,k
LiDi	ULT	20 s	Gr 10	0.500	0.067	f
LiDi	ULT	60 s	Gr 11	0.625	0.051	h,i
LiDi	ULT	30 m	Gr 12	0.711	0.053	j,k
Zir	ZiE	20 s	Gr 13	0.306	0.027	b,c,d,e
Zir	ZiE	60 s	Gr 14	0.335	0.031	c,d,e
Zir	ZiE	30 m	Gr 15	0.378	0.043	e
Zir	IPS	20 s	Gr 16	0.177	0.019	a
Zir	IPS	60 s	Gr 17	0.238	0.028	a,b
Zir	IPS	30 m	Gr 18	0.258	0.032	b,c
Zir	BISCO	20 s	Gr 19	0.245	0.034	a,b,c
Zir	BISCO	60 s	Gr 20	0.266	0.032	b,c,d
Zir	BISCO	30 m	Gr 21	0.271	0.030	b,c,d
Zir	ULT	20 s	Gr 22	0.245	0.035	a,b,c
Zir	ULT	60 s	Gr 23	0.296	0.029	b,c,d
Zir	ULT	30 m	Gr 24	0.314	0.039	d,e

*Note:* BIS = 9.5% Porcelain Etchant, Bisco; ULT = 9% Porcelain Etch, Ultradent; IPS = IPS Ceramic Etching Gel, Ivoclar).

Abbreviations: LiDi, lithium disilicate; Zir, zirconia.

### 3.1. Effect on LiDi

For the LiDi ceramic, all four etchants produced a substantial increase in surface roughness. However, Zircos‐E was again the most aggressive agent. It produced the highest Ra values at all time points, culminating in the highest roughness value of the entire study (Gr 3, Ra = 0.785 µm) after 30 min of application. This value was significantly higher than that produced by any other etchant at any time point on LiDi (*p*  < 0.05).

The traditional etchants (IPS, BIS, and ULT) created less roughness compared to Zircos‐E but were still highly effective on the silica‐based structure. At the 20‐s interval, which is clinically standard for LiDi, the Ra values were 0.535 µm (IPS), 0.573 µm (BIS), and 0.500 µm (ULT). There were no statistically significant differences among these three groups at the 20‐s mark. As etching time increased to 30 min, all traditional etchants showed a significant increase in the roughness they produced, but they remained statistically like one another in their effect.

### 3.2. Effect on 5‐Y Zir

On the Zir substrate, the novel multiacid etchant (Zircos‐E) demonstrated a significantly greater etching capability compared to the three traditional HF‐based etchant’s at all time points. The surface roughness for the Zircos‐E groups increased with application time, from 0.306 µm at 20 s to a maximum of 0.378 µm at 30 min. The 30‐min application of Zircos‐E (Gr 15) resulted in the highest surface roughness among all Zir groups, and this was statistically significant compared to all other Zir treatment groups (*p*  < 0.05).

The three traditional etchants (IPS, BIS, and ULT) produced much lower Ra values on Zir. The lowest roughness was observed with IPS etchant for 20 s (Gr 16, Ra = 0.177 µm). While there was a general trend of increasing roughness with time for these etchants, the differences were often not statistically significant among them. For instance, at the 20‐s interval, there was no significant difference between the BIS (Gr 19) and ULT (Gr 22) groups.

## 4. Discussion

### 4.1. General Considerations

Etching 5 mol% Y‐TZP (5Y‐TZP) remains challenging due to its high crystalline content and chemical inertness, unlike the easier HF etching of silica‐based ceramics. A multiacid solution (HF, HCl, H_2_SO_4_, HNO_3_, and H_3_PO_4_) combining complementary mechanisms has been proposed. HF initiates dissolution, HCl and HNO_3_ accelerate the process, H_2_SO_4_ enhances acidity, and H_3_PO_4_ improves surface chemistry. This synergy produces more uniform roughening than HF alone and avoids the microcracks associated with mechanical abrasion. Evidence shows that etching times up to 30–60 min can significantly increase roughness and bond strength without compromising hardness, while longer exposures may lead to saturation effects. These findings highlight the clinical relevance of multiacid etching, particularly for high‐translucency Zir, as it provides microporous surfaces favorable for resin bonding [20–23].

The three conventional HF etchants were ineffective on Zir but performed comparably on LiDi, creating clinically appropriate roughness at the 20‐s mark. As etching time increased, all etchants produced a rougher surface on both materials. The novel multiacid solution, Zircos‐E, proved highly effective on 5‐Y Zir, creating a significantly rougher surface than traditional etchants at all time points. Conversely, Zircos‐E was excessively aggressive on LiDi, resulting in extreme surface roughness indicative of structural damage.

The results of this investigation led to the rejection of both null hypotheses. The first null hypothesis, which posited that the final surface roughness would not be influenced by the type of ceramic material, was refuted. This was evidenced by the statistically significant difference observed between the two materials under study. The second hypothesis that the etchant and time combination would have no significant effect on surface roughness was also rejected, with the data clearly indicating that different etching protocols produced significantly different outcomes. Furthermore, the significant interaction between the main factors underscores that the etchants’ effects were dependent on the specific ceramic substrate, invalidating a universal etching approach.

### 4.2. Acid Etching as an Alternative to Sandblasting

From a clinical standpoint, combining airborne abrasion (sandblasting) with acid etching offers a great way to bond dental materials securely without compromising their long‐term strength. While sandblasting is great for initial bonding, it can cause microscopic changes to the material’s surface, which may weaken the bond over time. Acid etching, being a gentler process, creates a chemically stable surface that helps the bond last longer, especially when used with the right resin cements. The most significant finding of this investigation is the exceptional performance of Zircos‐E on 5‐Y Zir. Its unique multiacid formulation was capable of significantly altering the inert Zir surface, creating a degree of microroughness that was unattainable with the traditional HF etchants. However, the present investigation evaluated only surface roughness outcomes. Although increased roughness may contribute to micromechanical retention, it should not be interpreted as direct evidence of improved bond strength. Adhesive performance is influenced by multiple variables, including surface chemistry, wettability, resin infiltration, and chemical interactions with functional monomers. Therefore, additional studies evaluating bond strength and failure modes are required before definitive conclusions regarding adhesive performance can be established of a synergistic chemical action, where the combination of acids can effectively dissolve the crystalline grain boundaries of the Zir structure [24].

For silica‐based ceramics, using a lower concentration of HF, specifically around 5%, for a short duration of ~20 s, effectively prepares the surface without inducing excessive internal dissolution. Using higher concentrations (e.g., 10%) or prolonged etching times increases the risk of this internal damage and subsequent strength reduction. Higher HF concentrations and longer application times have been shown to enlarge these internal dissolution areas, leading to decreased strength and creating pathways for crack propagation that diminish the restoration’s durability [25].

### 4.3. Suitability of Multiacid Etching Solutions

To investigate the suitability of multiacid etching solutions for different materials, this study varied etching times, confirming that increased time significantly influences the surface roughness and microstructure of LiDi. Longer etching times generally increase surface roughness, but prolonged application can dissolve the glassy matrix and expose crystalline phases, leading to potential microstructural degradation. Excessive etching leads to overetching, which erodes the microstructure and reduces mechanical stability [26]. The elevated roughness values observed for Zircos‐E on LiDi may suggest a more aggressive interaction with the glassy matrix. Nevertheless, the present study did not evaluate flexural strength, fracture resistance, or microstructural damage. Therefore, the biological and mechanical significance of these roughness values should be interpreted with caution.

The clinical goal is to create sufficient roughness for bonding without weakening the material. For instance, overetching LiDi with 5% HF for 60 s results in larger crystals (~7 μm) compared to the finer crystals (~1 μm) produced by a standard 20‐s etch. Additionally, it is critical to ensure that etched surfaces are thoroughly cleaned, as residual precipitate salts can inhibit proper bonding if not completely removed. For Zir, the most durable bonds are achieved by combining mechanical abrasion with MDP‐containing primers [27].

### 4.4. Effects of Acid Etching on 5‐Y Zir

The long‐term stability of Zir restorations is challenged by low‐temperature degradation (LTD), a process where the material’s crystal structure changes from tetragonal to monoclinic, causing surface roughness and microcracking. According to the literature, deep etching of Zir can introduce surface flaws, microcracks, and residual stresses, which may act as initiation sites for LTD. This degradation process involves phase transformation and surface roughening, potentially compromising the long‐term stability and mechanical integrity of Zir restorations [28]. The presence of surface flaws from deep etching can accelerate LTD, making it a significant limitation and risk factor for the durability of Zir‐based restorations over time. A restoration’s resistance to this aging is tied to its microstructure and yttria content. While not a direct cause, acid etching may accelerate degradation by creating microscopic surface flaws that can serve as starting points for LTD [28].

Based on current research, an HF etching time of ~20 s using a 4.9% HF gel is generally optimal for LiDi ceramics. This duration effectively increases surface roughness for strong micromechanical retention without significantly weakening the material. It has been found that Ra values increase with longer etching, but these extended times also lead to a notable decrease in flexural strength, raising concerns about potential restoration failure. Therefore, exceeding 60 s is generally discouraged [29].

Clinicians should adhere to manufacturer recommendations to avoid overetching. Proper laboratory procedures include precise timing and thorough rinsing to remove residual acid. Using a neutralizing agent or applying silane after etching can further enhance bond strength. It is advisable to avoid prolonged etching beyond 20–30 s unless supported by specific evidence or manufacturer instructions. Achieving durable adhesion to high‐strength Zir has long been a clinical challenge. Conventional surface conditioning via airborne‐particle abrasion, while effective at increasing roughness, introduces iatrogenic surface flaws and microcracks that can compromise the material’s long‐term structural integrity.

Further investigations have revealed that synergistic effects can be achieved by combining this chemical priming with more controlled surface modification techniques. Novel acidic etching agents that alter the ceramic’s topography to create a microretentive pattern are ideal for interlocking. The efficacy of this integrated protocol is validated by significant increases in bond strength and a diagnostic shift in failure modes from predominantly adhesive to cohesive [30].

For LiDi, surface treatment with HF is essential for creating micromechanical retention. HF etching dissolves the glassy phase, exposing the crystalline needles of Li_2_Si_2_O_5_. However, this process can introduce residual fluorine‐containing compounds on the surface, which can interfere with subsequent bonding procedures if not properly cleaned [31].

Durable adhesion to silica‐based ceramics involves both micromechanical retention from etching and covalent chemical bonding. HF etching creates a microscopic, porous topography by dissolving the glassy phase matrix. While increased exposure generally enhances roughness, overetching is counterproductive, as it can weaken the ceramic substructure and paradoxically decrease bond strength [32].

The chemistry of this process involves HF reacting with the silica matrix to form volatile silicon tetrafluoride (SiF_4_) and then a soluble complex ion, hexafluorosilicate, which can be rinsed off. This removal of the glassy phase creates a deep porous structure. Research has shown that both etching time and HF concentration impact the resulting surface, with higher concentrations generally producing more uniform crystalline patterns [33].

Acidic environments can also pose a chemical challenge to Zir through yttrium ion leaching. Under acidic conditions, yttrium ions (Y^3+^) are more prone to release, which weakens the bonds in the Zir lattice. This process destabilizes the tetragonal phase, promoting its transformation to the monoclinic phase, which contributes to surface microcracking and degradation. This yttrium depletion is a key driver of microstructural changes that diminish the material’s mechanical integrity over time [34].

Excessive etching leads to overporosity and surface weakening. For LiDi, initiating etching at 20 s is recommended, as it was suggested that this duration maximizes bond strength while minimizing damage. Establishing a standardized etching time of 20 s with a 5%–10% HF solution is a reliable way for clinicians to ensure the surface has a suitable microporosity for bonding [35].

Other surface treatments also have profound effects on Zir. Coarser grinding protocols can increase roughness but also create microcracks, while fine polishing can restore surface smoothness. These treatments can induce residual stresses and promote phase transformations that may lead to aging and degradation. The longevity of Zir restorations, therefore, depends heavily on employing proper surface treatment protocols that optimize smoothness and phase stability.

The concentration and duration of HF treatment significantly influence the surface roughness of CAD/CAM ceramics. Higher HF concentrations and longer etching times tend to increase surface roughness, which can enhance mechanical interlocking. However, excessive etching may cause surface damage and compromise the ceramic’s integrity. The long‐term durability needs to be investigated, since HF etching, while initially producing higher surface roughness, may lead to surface degradation over time [36].

The results for 9% HF etching include increased surface roughness. This agrees with the findings of Porto et al. that found increased surface roughness after 30 s of application, while for 90 s of application, deeper microporosity as increased surface roughness was evident. These findings indicate that longer etching times result in deeper and more extensive surface modifications [37].

Etching with Zircos–E creates a more uniform and rougher surface, which enhances micromechanical bonding, showing that this method results in higher microshear bond strength compared to other surface treatments, especially when combined with air abrasion in translucent Zir. However, air abrasion alone caused some material loss and surface irregularities [38].

In spite seems to be merely a descriptive parameter, surface roughness is a well‐established indicator of structural alterations in ceramic substrates. Profilometry provides quantitative, statistically significant evidence of overetching, and prior studies consistently demonstrate that excessive roughness correlates with microstructural weakening of LiDi. In the present work, the markedly higher Ra values are observed with Zircos‐E, which is in line with findings reported in the literature, even in the absence of direct scanning electron microscopy (SEM) imaging. In addition, the experimental design included four different etchants, two ceramic systems, and three application times, yielding a comprehensive interaction matrix. This approach revealed the novel and clinically relevant finding that the same etchant may enhance Zir surfaces but cause damage to LiDi [39]. Future studies should investigate bond strength, surface morphology, phase stability, and long‐term durability to determine the clinical relevance of the surface modifications observed in the present study.

A major limitation of this study is that surface characterization was restricted to the Ra roughness parameter obtained by contact profilometry. Although Ra is a widely accepted indicator of surface modification, it does not fully describe the complexity of surface morphology or predict adhesive behavior. Additional characterization methods, including SEM, atomic force microscopy (AFM), surface energy analysis, phase transformation evaluation, and bond strength testing, would provide a more comprehensive understanding of the effects produced by the evaluated etchants.

## 5. Conclusion

Within the limitations of this in vitro study, the following conclusions can be drawn:

1. The novel multiacid etchant (Zircos‐E) produced significantly higher surface roughness values on 5Y‐TZP Zir compared with conventional HF etchants. Further studies evaluating bond strength, surface morphology, phase transformation, and long‐term durability are required before clinical recommendations can be established.
2. On LiDi, the multiacid etchant generated substantially higher roughness values than conventional HF etchants. The clinical implications of this finding remain uncertain and require further investigation through mechanical and adhesive testing.
3. Conventional HF etchants produced similar roughness values on LiDi and limited surface modification on Zir.

## Author Contributions

Conceptualization: Silvia Rojas‐Rueda, Fabio Andretti, and Abdulrahman Alshabib. Methodology: Carlos A. Jurado, Hamad Algamaiah, Franciele Floriani, and Mark Adam Antal. Software: Hamad Algamaiah and Mark Adam Antal. Validation: Franciele Floriani, Fabio Andretti, Carlos A. Jurado, and Abdulrahman Alshabib. Formal analysis: Silvia Rojas‐Rueda and Fabio Andretti. Investigation: Abdulrahman Alshabib and Hamad Algamaiah. Resources: Franciele Floriani and Mark Adam Antal. Data curation: Silvia Rojas‐Rueda and Mark Adam Antal. Writing – original draft preparation: Fabio Andretti and Abdulrahman Alshabib. Writing – review and editing: Silvia Rojas‐Rueda, Hamad Algamaiah, and Mark Adam Antal. Visualization: Franciele Floriani and Fabio Andretti. Supervision: Abdulrahman Alshabib, Mark Adam Antal, and Carlos A. Jurado. Project administration: Franciele Floriani, Hamad Algamaiah, and Abdulrahman Alshabib. Funding acquisition: Abdulrahman Alshabib and Silvia Rojas‐Rueda.

## Funding

This study did not receive any specific funding from public, commercial, or not‐for‐profit funding agencies.

## Disclosure

All authors have read and approved the final version of the manuscript. Franciele Floriani, as the corresponding author, had full access to all of the data in this study and takes complete responsibility for the integrity of the data and the accuracy of the data analysis. All authors contributed to the revision and final approval of the manuscript.

## Conflicts of Interest

The authors declare no conflicts of interest.

## Data Availability

The authors confirm that the data supporting the findings of this study are available within the article.

## References

[1] Ghodsi S. , Arzani S. , Shekarian M. , and Aghamohseni M. , Cement Selection Criteria for Full Coverage Restorations: A Comprehensive Review of Literature, Journal of Clinical and Experimental Dentistry. (2021) 13, e1154–e1161, 10.4317/jced.58671. 34824703 PMC8601696

[2] Azevedo V. L. B. , de Castro E. F. , and Bonvent J. J. , et al.Surface Treatments on CAD/CAM Glass–Ceramics: Influence on Roughness, Topography, and Bond Strength, Journal of Esthetic and Restorative Dentistry. (2021) 33, no. 5, 739–749, 10.1111/jerd.12734. 33991436

[3] Gracis S. , Thompson V. P. , Ferencz J. L. , Silva N. R. F. A. , and Bonfante E. A. , A New Classification System for All-Ceramic and Ceramic-Like Restorative Materials, The International Journal of Prosthodontics. (2016) 28, no. 3, 227–235, 10.11607/ijp.4244. 25965634

[4] Cuzic C. , Rominu M. , and Pricop A. , et al.Clinician’s Guide to Material Selection for All-Ceramics in Modern Digital Dentistry, Materials. (2025) 18, no. 10, 10.3390/ma18102235, 2235. 40428971 PMC12113582

[5] Fu L. , Engqvist H. , and Xia W. , Glass–Ceramics in Dentistry: A Review, Materials. (2020) 13, no. 5, 10.3390/ma13051049, 1049. 32110874 PMC7084775

[6] Streit G. and Sykes L. M. , Overview of Lithium Disilicate as a Restorative Material in Dentistry, South African Dental Journal. (2022) 77, no. 8, 495–499, 10.17159/2519-0105/2022/v77no8a7.

[7] Sundfeld-Neto D. , Naves L. Z. , and Costa A. R. , et al.The Effect of Hydrofluoric Acid Concentration on the Bond Strength and Morphology of the Surface and Interface of Glass Ceramics to a Resin Cement, Operative Dentistry. (2015) 40, 470–479, 10.2341/14-133-L. 25764043

[8] Murillo-Gómez F. , Hernández-Víquez J. R. , and Sauma-Montes de Oca J. R. , et al.Mechanical, Adhesive and Surface Properties of a Zirconia-Reinforced Lithium Silicate CAD/CAM Ceramic Exposed to Different Etching Protocols, Materials. (2024) 17, no. 20, 10.3390/ma17205039, 5039. 39459744 PMC11509709

[9] Denry I. and Kelly J. R. , State of the Art of Zirconia for Dental Applications, Dental Materials. (2008) 24, no. 3, 299–307, 10.1016/j.dental.2007.05.007. 17659331

[10] Bebsh M. , Haimeur A. , and França R. , The Effect of Different Surface Treatments on the Micromorphology and the Roughness of Four Dental CAD/CAM Lithium Silicate-Based Glass-Ceramics, Ceramics. (2021) 4, no. 3, 467–475, 10.3390/ceramics4030034.

[11] Veríssimo A. H. , Moura D. M. D. , Tribst J. P. M. , Araújo A. M. M. , Leite F. P. P. , and Souza R. O. A. , Effect of Hydrofluoric Acid Concentration and Etching Time on Resin-Bond Strength to Different Glass Ceramics, Brazilian Oral Research. (2019) 33, 10.1590/1807-3107bor-2019.vol33.0041. 31508723

[12] Rao H. M. , Kumaraswamy M. , Thomas D. , Boraiah S. , and Rana K. S. , Zirconia in Restorative Dentistry, Zirconia–New Advances, Structure, Fabrication and Applications, IntechOpen, 2023 10.5772/intechopen.111601.

[13] Kelly J. R. and Denry I. , Stabilized Zirconia as a Structural Ceramic: An Overview, Dental Materials. (2008) 24, no. 3, 289–298, 10.1016/j.dental.2007.05.005. 17624420

[14] Bacchi A. and Cesar P. F. , Advances in Ceramics for Dental Applications, Dental Clinics of North America. (2022) 66, 591–602, 10.1016/j.cden.2022.05.007. 36216448

[15] D’Alessandro C. , Josic U. , and Mazzitelli C. , et al.Is Zirconia Surface Etching a Viable Alternative to Airborne Particle Abrasion? A Systematic Review and Meta-Analysis of In Vitro Studies, Journal of Dentistry. (2024) 151, 10.1016/j.jdent.2024.105394, 105394. 39374733

[16] Yahyazadehfar N. , Azimi Zavaree M. , Shayegh S. S. , Yahyazadehfar M. , Hooshmand T. , and Hakimaneh S. M. R. , Effect of Different Surface Treatments on Surface Roughness, Phase Transformation, and Biaxial Flexural Strength of Dental Zirconia, Journal of Dental Research, Dental Clinics, Dental Prospects. (2021) 15, 210–218, 10.34172/joddd.2021.035. 34712413 PMC8538149

[17] Hallmann L. , Ulmer P. , and Wille S. , et al.Effect of Surface Treatments on the Properties and Morphological Change of Dental Zirconia, The Journal of Prosthetic Dentistry. (2016) 115, no. 3, 341–349, 10.1016/j.prosdent.2015.09.007. 26581661

[18] Chevalier J. , Gremillard L. , Virkar A. V. , and Clarke D. R. , The Tetragonal-Monoclinic Transformation in Zirconia: Lessons Learned and Future Trends, Journal of The American Ceramic Society. (2009) 92, no. 9, 1901–1920, 10.1111/j.1551-2916.2009.03278.x.

[19] Conner C. , Andretti F. , and Hernandez A. I. , et al.Surface Evaluation of a Novel Acid-Etching Solution for Zirconia and Lithium Disilicate, Materials. (2025) 18, no. 12, 10.3390/ma18122912, 2912. 40573041 PMC12195062

[20] Kim S.-H. , Oh K. C. , and Moon H.-S. , Effects of Surface-Etching Systems on the Shear Bond Strength of Dual-Polymerized Resin Cement and Zirconia, Materials. (2024) 17, no. 13, 10.3390/ma17133096, 3096. 38998179 PMC11242500

[21] Smielak B. and Klimek L. , Effect of Hydrofluoric Acid Concentration and Etching Duration on Select Surface Roughness Parameters for Zirconia, The Journal of Prosthetic Dentistry. (2015) 113, no. 6, 596–602, 10.1016/j.prosdent.2015.01.001. 25799283

[22] Cho J. H. , Kim S. J. , Shim J. S. , and Lee K.-W. , Effect of Zirconia Surface Treatment Using Nitric Acid-Hydrofluoric Acid on the Shear Bond Strengths of Resin Cements, The Journal of Advanced Prosthodontics. (2017) 9, no. 2, 77–84, 10.4047/jap.2017.9.2.77. 28435615 PMC5397592

[23] Ansari S. , Jahedmanesh N. , and Cascione D. , et al.Effects of an Etching Solution on the Adhesive Properties and Surface Microhardness of Zirconia Dental Ceramics, The Journal of Prosthetic Dentistry. (2018) 120, no. 3, 447–453, 10.1016/j.prosdent.2017.11.026. 29703674

[24] Andretti F. , Jurado C. A. , and Antal M. , et al.Surface Assessment of a Novel Acid-Etching Solution on CAD/CAM Dental Ceramics, Biomimetics. (2025) 10, no. 8, 10.3390/biomimetics10080508, 508. 40862881 PMC12383666

[25] Leyva D. R. D. , Sandoval-Sanchez E. , Campos-Villegas N. E. , Azpiazu-Flores F. X. , and Zavala-Alonso N. V. , Influence of Heated Hydrofluoric Acid Surface Treatment on Surface Roughness and Bond Strength to Feldspathic Ceramics and Lithium-Disilicate Glass-Ceramics, Journal of Adhesive Dentistry. (2021) 23, no. 6, 549–555, 10.3290/j.jad.b2288275. 34817970

[26] Garfias C. S. and de Goes M. F. , Acid Dissolution of Ultrathin Glass-Ceramic and Its Correlation With Flexural Strength, The Journal of Prosthetic Dentistry. (2022) 128, no. 5, 1084.e1–1084.e8, 10.1016/j.prosdent.2022.08.035. 36460426

[27] Phark J. H. and Duarte S.Jr, Microstructural Considerations for Novel Lithium Disilicate Glass Ceramics: A Review, Journal of Esthetic and Restorative Dentistry. (2022) 34, no. 1, 92–103, 10.1111/jerd.12864. 34995008

[28] Benalcázar-Jalkh E. B. , Bergamo E. T. P. , and Campos T. M. B. , et al.A Narrative Review on Polycrystalline Ceramics for Dental Applications and Proposed Update of a Classification System, Materials. (2023) 16, no. 24, 10.3390/ma16247541, 7541. 38138684 PMC10744432

[29] Zogheib L. V. , Della Bona A. , Kimpara E. T. , and McCabe J. F. , Effect of Hydrofluoric Acid Etching Duration on the Roughness and Flexural Strength of a Lithium Disilicate-Based Glass Ceramic, Brazilian Dental Journal. (2011) 22, no. 1, 45–50, 10.1590/S0103-64402011000100008. 21519648

[30] Zhu H. , Chiang C. C. , Craciun V. , Deane G. M. , Ren F. , and Esquivel-Upshaw J. F. , Yttrium Ion Release and Phase Transformation in Yttria-Stabilized Zirconia Under Acidic Conditions: Implications for Dental Implant Durability, Materials (Basel). (2025) 18, no. 14, 10.3390/ma18143311, 3311. 40731521 PMC12300729

[31] Ho G. W. and Matinlinna J. P. , Insights on Ceramics as Dental Materials. Part II: Chemical Surface Treatments, Silicon. (2011) 3, no. 3, 117–123, 10.1007/s12633-011-9079-6.

[32] Ramakrishnaiah R. , Alkheraif A. A. , Divakar D. D. , Matinlinna J. P. , and Vallittu P. K. , The Effect of Hydrofluoric Acid Etching Duration on the Surface Micromorphology, Roughness, and Wettability of Dental Ceramics, International Journal of Molecular Sciences. (2016) 17, no. 6, 10.3390/ijms17060822, 822. 27240353 PMC4926356

[33] Botelho M. G. , Dangay S. , Shih K. , and Lam W. Y. H. , The Effect of Surface Treatments on Dental Zirconia: An Analysis of Biaxial Flexural Strength, Surface Roughness and Phase Transformation, Journal of Dentistry. (2018) 75, 65–73, 10.1016/j.jdent.2018.05.016. 29842902

[34] Almirabi R. S. and Alzahrani K. M. , Effect of the Intaglio Surface Treatment and Thickness of Different Types of Yttria-Stabilized Tetragonal Zirconia Polycrystalline Materials on the Flexural Strength: In-Vitro Study, Materials. (2024) 17, no. 21, 10.3390/ma17215256, 5256. 39517532 PMC11548017

[35] Poulon-Quintin A. , Ogden E. , and Large A. , et al.Chemical Surface Modification of Lithium Disilicate Needles of a Silica-Based Ceramic After HF-Etching and Ultrasonic Bath Cleaning: Impact on the Chemical Bonding With Silane, Dental Materials. (2021) 37, no. 5, 832–839, 10.1016/j.dental.2021.02.006. 33640173

[36] Oliveira L. T. , de Castro E. F. , and Azevedo V. L. B. , et al.Effect of Ceramic Conditioners on Surface Morphology, Roughness, Contact Angle, Adhesion, Microstructure, and Composition of CAD/CAM Ceramics, Operative Dentistry. (2023) 48, 277–293, 10.2341/21-078-L. 36929774

[37] Porto T. S. , da Silva I. G. M. , Vallerini B. F. , and de Goes M. F. , Different Surface Treatment Strategies on Etchable CAD-CAM Materials: Part 1-Effect on the Surface Morphology, The Journal of Prosthetic Dentistry. (2023) 130, no. 5, 761–769, 10.1016/j.prosdent.2021.10.020. 35094771

[38] Sales A. , Rodrigues S. J. , and Mahesh M. , et al.Effect of Different Surface Treatments on the Micro-Shear Bond Strength and Surface Characteristics of Zirconia: An In Vitro Study, International Journal of Dentistry. (2022) 2022, no. 1, 10.1155/2022/1546802, 1546802. 35464102 PMC9023206

[39] Dos Santos D. , Pacheco R. R. , and Komegae G. H. , et al.Effects of Hydrofluoric Acid Concentrations, Commercial Brands, and Adhesive Application on the Bond Strength of a Resin Luting Agent to Lithium Disilicate Glass Ceramic, Operative Dentistry. (2023) 48, no. 6, 700–710, 10.2341/23-034-L. 37881098

